# Decreased β-cell volume and insulin secretion but preserved glucose tolerance in a growth hormone insensitive pig model

**DOI:** 10.1007/s11102-024-01424-w

**Published:** 2024-07-03

**Authors:** Laeticia Laane, Simone Renner, Elisabeth Kemter, Michael Stirm, Birgit Rathkolb, Andreas Blutke, Martin Bidlingmaier, Martin Hrabĕ de Angelis, Eckhard Wolf, Arne Hinrichs

**Affiliations:** 1 Chair for Molecular Animal Breeding and Biotechnology, Gene Center and Department of Veterinary Sciences, LMU Munich, Munich, Germany; 2 Center for Innovative Medical Models (CiMM), LMU Munich, Oberschleißheim, Germany; 3 German Center for Diabetes Research (DZD), Neuherberg, Germany; 4 Institute of Experimental Genetics, German Mouse Clinic (GMC), Helmholtz Zentrum München, Neuherberg, Germany; 5 Institute of Veterinary Pathology, Center for Clinical Veterinary Medicine, LMU Munich, Munich, Germany; 6 Endocrine Laboratory, Medizinische Klinik Und Poliklinik IV, Klinikum Der Universität München, Munich, Germany; 7 Chair of Experimental Genetics, School of Life Science Weihenstephan, Technische Universität München, Freising, Germany; 8 Interfaculty Center for Endocrine and Cardiovascular Disease Network Modelling and Clinical Transfer (ICONLMU), LMU Munich, Munich, Germany

**Keywords:** β-cell, Growth hormone insensitivity, Pig model, Insulin sensitivity, Glucose tolerance, Quantitative stereology

## Abstract

**Purpose:**

Growth hormone (GH) is a central regulator of β-cell proliferation, insulin secretion and sensitivity. Aim of this study was to investigate the effect of GH insensitivity on pancreatic β-cell histomorphology and consequences for metabolism in vivo.

**Methods:**

Pancreata from pigs with growth hormone receptor deficiency (*GHR*-KO, n = 12) were analyzed by unbiased quantitative stereology in comparison to wild-type controls (WT, n = 12) at 3 and 7–8.5 months of age. In vivo secretion capacity for insulin and glucose tolerance were assessed by intravenous glucose tolerance tests (ivGTTs) in *GHR*-KO (n = 3) and WT (n = 3) pigs of the respective age groups.

**Results:**

Unbiased quantitative stereological analyses revealed a significant reduction in total β-cell volume (83% and 73% reduction in young and adult *GHR*-KO vs. age-matched WT pigs; p < 0.0001) and volume density of β-cells in the pancreas of *GHR*-KO pigs (42% and 39% reduction in young and adult *GHR*-KO pigs; p = 0.0018). *GHR*-KO pigs displayed a significant, age-dependent increase in the proportion of isolated β-cells in the pancreas (28% in young and 97% in adult *GHR*-KO vs. age-matched WT pigs; p = 0.0009). Despite reduced insulin secretion in ivGTTs, *GHR*-KO pigs maintained normal glucose tolerance.

**Conclusion:**

GH insensitivity in *GHR*-KO pigs leads to decreased β-cell volume and volume proportion of β-cells in the pancreas, causing a reduced insulin secretion capacity. The increased proportion of isolated β-cells in the pancreas of *GHR*-KO pigs highlights the dependency on GH stimulation for proper β-cell maturation. Preserved glucose tolerance accomplished with decreased insulin secretion indicates enhanced sensitivity for insulin in GH insensitivity.

**Supplementary Information:**

The online version contains supplementary material available at 10.1007/s11102-024-01424-w.

## Introduction

Growth hormone (GH) is a central regulator of longitudinal growth and development. GH stimulates β-cell proliferation, insulin (*INS*) gene transcription and insulin secretion [1–3], but also has direct effects on metabolism [4], acting as an antagonist of insulin action [5] and stimulating lipolysis [6]. Accordingly, in conditions of GH insensitivity due to deficiency of the growth hormone receptor (human Laron syndrome [7]), growth retardation and an increased accumulation of adipose tissue are displayed [8, 9]. In human patients with Laron syndrome from an Ecuadorian cohort, a decreased proportion of β-cells in the pancreas was assumed from a significantly reduced homeostasis model assessment of β-cell function (HOMA-β score). Nevertheless, while those patients generally showed decreased serum insulin levels and a reduced insulin secretion in glucose tolerance tests, their glucose tolerance was preserved, suggesting an improved sensitivity to insulin in the absence of GH action [10]. Studies on pancreata from the murine model for GH insensitivity (the *Ghr*-KO mouse) revealed a decrease in both, absolute and relative islet cell mass, along with a reduction in islet cell size. Contrary to findings in the human cohort, the reduced insulin secretion capacity in *Ghr*-KO mice resulted in disturbed glucose tolerance [11]. The *GHR*-KO pig was recently developed as a large animal model for GH insensitivity and closely resembles the human phenotype regarding endocrine alterations [12], postnatal growth retardation and obesity [13, 14] as well as hallmarks of GH insensitivity-associated metabolism [14–16]. In this study, we demonstrate that a lack of GH action results in reduced β-cell mass and β-cell proportion in the pancreas, impaired maturation of β-cells and decreased insulin secretion capacity. Nevertheless, the absence of insulin-antagonizing GH action led to undisturbed glucose tolerance, similar to findings in the Ecuadorian cohort of human Laron syndrome patients, who display an increased insulin sensitivity despite obesity.

## Materials and methods

### Animals

The *GHR*-KO mutation was introduced by CRISPR/Cas9 in cultured cells and *GHR*-KO founder pigs were generated by somatic cell nuclear transfer as described previously [14]. Pigs used in this study were generated by mating sows and boars heterozygous for the *GHR* gene mutation. The groups consisted of pigs homozygous for the *GHR* mutation *(GHR*-KO pigs) and wild-type (WT) littermate control animals. All animal experiments were performed in accordance with the German Animal Welfare Act and the Directive 2010/63/EU on the protection of animals used for scientific purposes. All animal procedures performed were approved by the responsible animal welfare authority (Regierung von Oberbayern, permissions ROB-55.2-1-54-2532-70-12, ROB-55.2Vet-2532.Vet_02-17-136 and ROB-55.2Vet-2532.Vet_02-22-92).

### Necropsy, histology and pancreas sampling

**Fig. 1:**
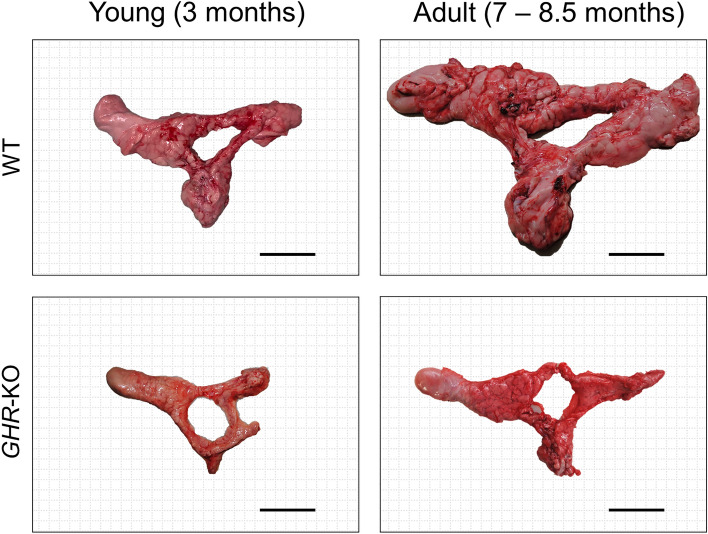
Size comparison of fresh pancreata from *GHR*-KO and wild-type (WT) pigs at young and adult age. Bar = 5 cm

Pancreata were obtained from *GHR*-KO (n = 12) and WT controls (n = 12) at either young (3 months; n = 5 vs. n = 5, all female) or adult age (7–8.5 months; n = 7 vs. n = 7, 3 males vs. 4 females each). Pigs were anesthetized by intravenous injection of ketamine (Ursotamin®, Serumwerk Bernburg) and xylazine (Xylazin 2%, Serumwerk Bernburg) followed by fentanyl (Fentadon®, Dechra) application. Subsequent to exsanguination, organs were sampled for further examination. After removal from the animal carcass and cleaning of connective tissue, the pancreas was weighed to the nearest g and photographed (Fig. 1). Representative samples of the pancreas were systematically and randomly sampled (as described in [17]). After cutting to size, tissue pieces were prefixed in 4% neutrally buffered formaldehyde solution for 24 h at room temperature, placed in embedding cassettes, routinely processed with a tissue processing system and embedded in paraffin. Microscopic sections (nominal thickness: 3 μm) were cut from paraffin blocks using a Microm HM 325 rotary microtome.

### Immunohistochemical detection of insulin

Insulin-positive cells in the pancreas were detected via immunohistochemistry according to Pilz et al. [18]. Briefly, after dewaxing, heat-induced antigen retrieval in citrate buffer (pH 6.0) for 15 min at sub boiling temperature, and endogenous peroxidase block by 1% H_2_O_2_, a mouse anti-insulin monoclonal antibody was used as the primary antibody (#I2018, Sigma; 1:3000) for overnight incubation at 4 °C, followed by biotinylated goat anti-mouse IgG (H+L) secondary antibody (#115-065-146, Jackson ImmunoResearch Laboratories, Inc.; 1:250 + 3% porcine serum) for 1 h at room temperature, and avidin–biotin complex (#PK-6100, Vector Laboratories) for 30 min at room temperature. A horseradish peroxidase DAB substrate kit (#SK-4100, Vector Laboratories) was used to detect bound antibodies. Nuclear counterstaining was performed with Meyer’s hemalum solution.

### Unbiased quantitative stereological analyses

The volume density of immunohistochemically insulin-positive cells within the pancreas (V_v(β-cells/Pan)_) was estimated by unbiased quantitative stereological analysis. An Olympus BX41 light microscope in combination with a connected camera (Olympus DP 72) and the newCAST™ stereology software (Visiopharm Integrator System, Visiopharm, version 3.6.2.0.) was used for the evaluation. Volume densities were determined using the point counting method. Points hitting the targeted structure’s sections were divided by those hitting the reference compartment within the same sections [19, 20]. The average number of systematically randomly sampled fields of view amounted to 233.7 ± 28.47 per case. On the average, 934.7 ± 113.9 points for pancreatic tissue and 23,367 ± 2846 points for β-cells were counted per case (at 200×magnification). Isolated β-cells were defined as individual or small clusters of insulin-positive cells (up to five nuclear profiles). The volume densities of β-cells (V_V(β-cell/Pan)_) and isolated β-cells (V_V(isoβ-cell/Pan)_) as well as the total volumes of β-cells (V_(β-cell, Pan)_) and of isolated β-cells (V_(isoβ-cell, Pan)_) in the pancreas were calculated according to [21, 22]. The terms “total volume” and “mass” are used synonymously. The specific weight of the pig pancreas (1.07 g/cm^3^) [21] was determined by the submersion method [23–25].

### Intravenous glucose tolerance tests

Insulin secretion capacity and glucose tolerance were assessed by intravenous glucose tolerance tests in *GHR*-KO pigs and WT controls at either age group (n = 3 per genotype and age). For glucose injection and blood withdrawal for insulin and glucose measurements, the pigs obtained central venous catheters (CareFlow™, Merit Medical®, size 2.5 or 3 French) into a marginal ear vein [26]. Pigs were fasted overnight (for 16 h) and afterwards attained a glucose bolus injection of 50% glucose solution (0.5 g/kg BW) though the central venous catheter. Blood samples were taken at the indicated time points before (− 10 and 0 min) and after glucose bolus injection (3, 5, 7, 10, 15, 20, 30, 40, 50, 60, 90 and 120 min).

### Metabolite and hormone assays

Serum Insulin-like growth factor 1 (IGF1) concentrations were measured using the iSYS automated chemiluminescent IGF1 assay (Immunodiagnostic Systems) as described previously [27]. Blood glucose levels were immediately determined in duplicate from freshly collected full blood using a FreeStyle Freedom Lite blood glucose meter (Abbott) and FreeStyle Lite blood glucose test strips (Abbott) [28]. After clotting for 30 min at room temperature and centrifugation (1200×*g*, 20 min, 4 °C), aliquots of serum were stored at − 80 °C. Insulin levels were determined from serum with a sandwich chemiluminescence immunoassay (LIAISON® Insulin, DiaSorin) in combination with a fully automated immunoassay analyzer (LIAISON® XL, DiaSorin). Non-esterified free fatty acids (NEFAs) were analyzed from EDTA plasma using an AU480 clinical chemistry analyzer (Beckman Coulter) and adapted reagent kits from FUJIFILM Wako Chemicals GmbH as described previously [29].

### Statistics

All data are displayed as mean ± SEM. PROC GLM (General Linear Models, SAS 8.2) was used to analyze the body weight at the day of necropsy, the area under the curve (AUC) values for glucose and insulin and all assessed parameters concerning quantitative stereology of the pancreas. Effects of group (*GHR*-KO, WT), age (young, adult), sex (male and female adults) and the interaction group*age were taken into consideration. None of the investigated parameters was significantly affected by sex. Results of ivGTTs (glucose and insulin levels) were analyzed using PROC MIXED (Linear Mixed Models; SAS 8.2) considering effects of group (*GHR*-KO, WT), age (young, adult) as well as the interaction group*age. The Graph Pad Prism software (version 5.02 and 10.1.2) was used to generate figures and calculate AUC values, means and SEMs. P values < 0.05 were defined as significant.

## Results

### Growth retardation, endocrine and metabolic alterations in *GHR*-KO pigs

**Fig. 2:**
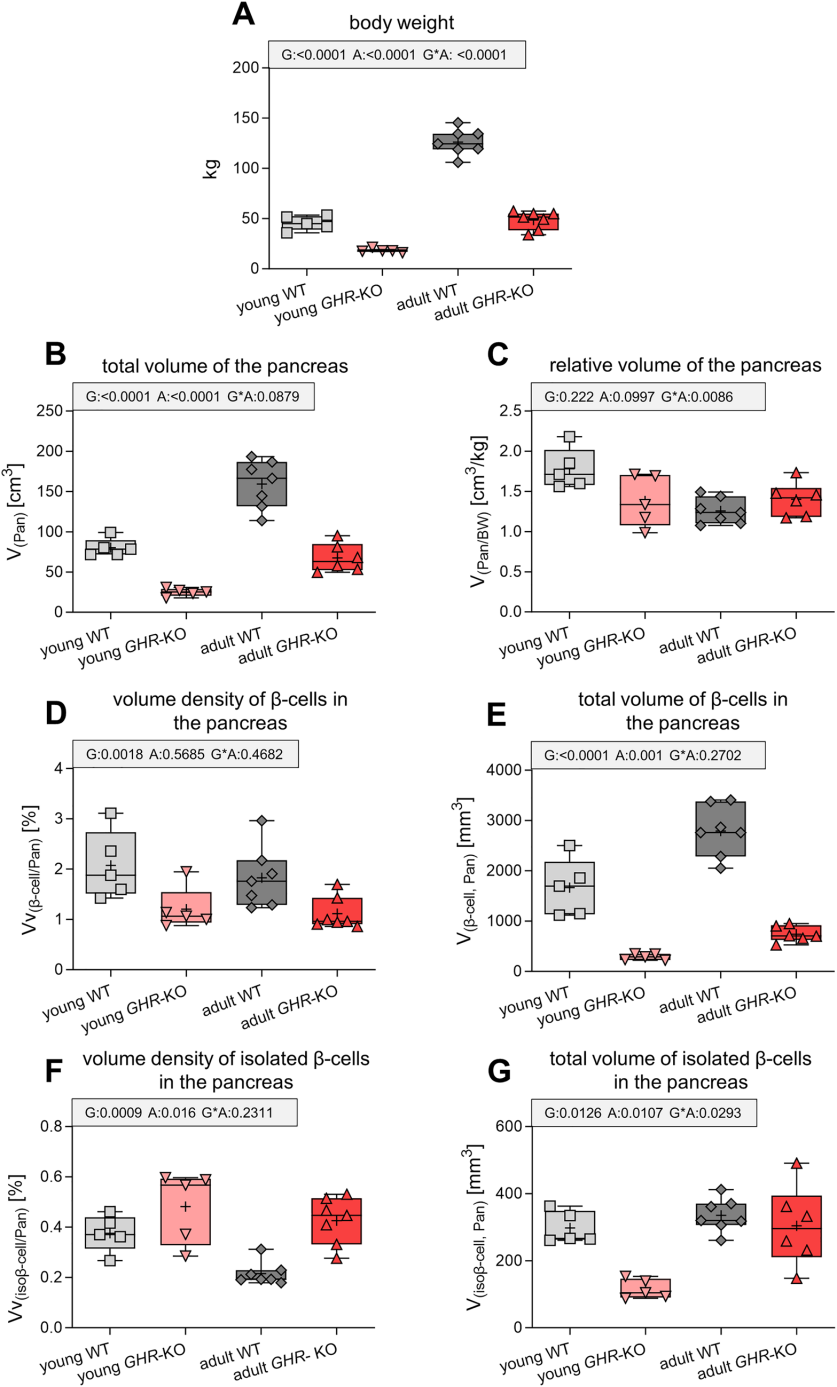
*GHR*-KO pigs displayed significantly reduced body weights (**A**) and pancreas volumes (**B**). The reduction in pancreas volumes appeared proportionally reduced with the reduction in body weights (**C**). The volume density of β-cells (**D**) as well as the total volume of β-cells in the pancreas (**E**) were significantly reduced in *GHR*-KO vs. WT control pigs regardless of age. A significant increase in volume density of isolated β-cells was observed in the pancreas from *GHR*-KO pigs, while a decrease with age was prominent in WT controls (**F**). The reduction in total volumes of isolated β-cells in *GHR*-KO pigs` pancreata was less pronounced in the adult group (**G**). The box plots show medians, 25th and 75th percentiles (box) and extremes (whiskers). The mean is marked as “ + ”. Abbreviations: G = group, A = age, G*A = interaction group*age

*GHR*-KO pigs displayed significantly reduced serum levels of IGF1 (Table 1; p = 0.0003 for the effect of group). Regardless of age, *GHR*-KO pigs showed a significant, ~ 60% reduction of body weight (Fig. 2A; Table S1; p < 0.0001 for the effect of group) as compared to WT controls (18.2 ± 0.9 kg vs. 45.6 ± 3.2 kg at young and 48.7 ± 3.4 kg vs. 126.2 ± 4.9 kg at adult age). Fasting insulin levels were significantly decreased in *GHR*-KO pigs (Table 1; p < 0.0001 for the effect of group). Fasting insulin levels increased with age in WT pigs from 6.2 ± 0.5 µIU/mL in young to 12.8 ± 3.5 µIU/mL in adult animals (p = 0.0001 for the effect of age) and even more in *GHR*-KO pigs from 0.5 ± 0.1 µIU/mL in young to 1.7 ± 0.3 µIU/mL in adults, although the absolute insulin levels were markedly lower in *GHR*-KO vs. WT pigs (p = 0.0006 for the interaction group*age). Fasting glucose levels were generally lower in *GHR*-KO than WT pigs (Table 1; p = 0.0005 for the effect of group) but were closer to WT pigs` values at adult age (49.3 ± 0.9 mg/dL in *GHR*-KO vs. 61.6 ± 2.3 mg/dL in WT pigs; p = 0.0321 for the interaction group*age). NEFA levels of young *GHR*-KO pigs were not different from those of age-matched controls (0.39 ± 0.04 mmol/L in young *GHR*-KO vs. 0.5 ± 0.02 mmol/L in young WT pigs), but slightly reduced at adult age (0.23 ± 0.05 mmol/L in adult *GHR*-KO vs. 0.45 ± 0.18 mmol/L in adult WT pigs; Table 1).

**Table 1:** Fasting IGF1, insulin, glucose and NEFA levels in *GHR*-KO pigs (young n = 3; adult n = 3) and WT controls (young n = 3; adult n = 3) aged 3 months (young) and 8.5 months (adult)

Parameter	Young WT	Young *GHR*-KO	Adult WT	Adult *GHR*-KO	Group	Age	Group*Age
	Mean ± SEM				p-value		
IGF1 (ng/mL)	255.7 ± 46.4	16 ± 1.7	258.7 ± 19.8	20 ± 2.1	0.0003	0.6067	0.5343
Insulin (µIU/mL)	6.2 ± 0.5	0.5 ± 0.1	12.8 ± 3.5	1.7 ± 0.3	< 0.0001	0.0001	0.0006
Glucose (mg/dL)	65.9 ± 1.7	43.8 ± 1.6	61.6 ± 2.3	49.3 ± 0.9	0.0005	0.8364	0.0321
NEFA (mmol/L)	0.5 ± 0.02	0.39 ± 0.04	0.45 ± 0.18	0.23 ± 0.05	0.2928	0.4817	0.6941

### β-cell mass and volume density of β-cells are decreased in *GHR*-KO pig pancreata

**Fig. 3:**
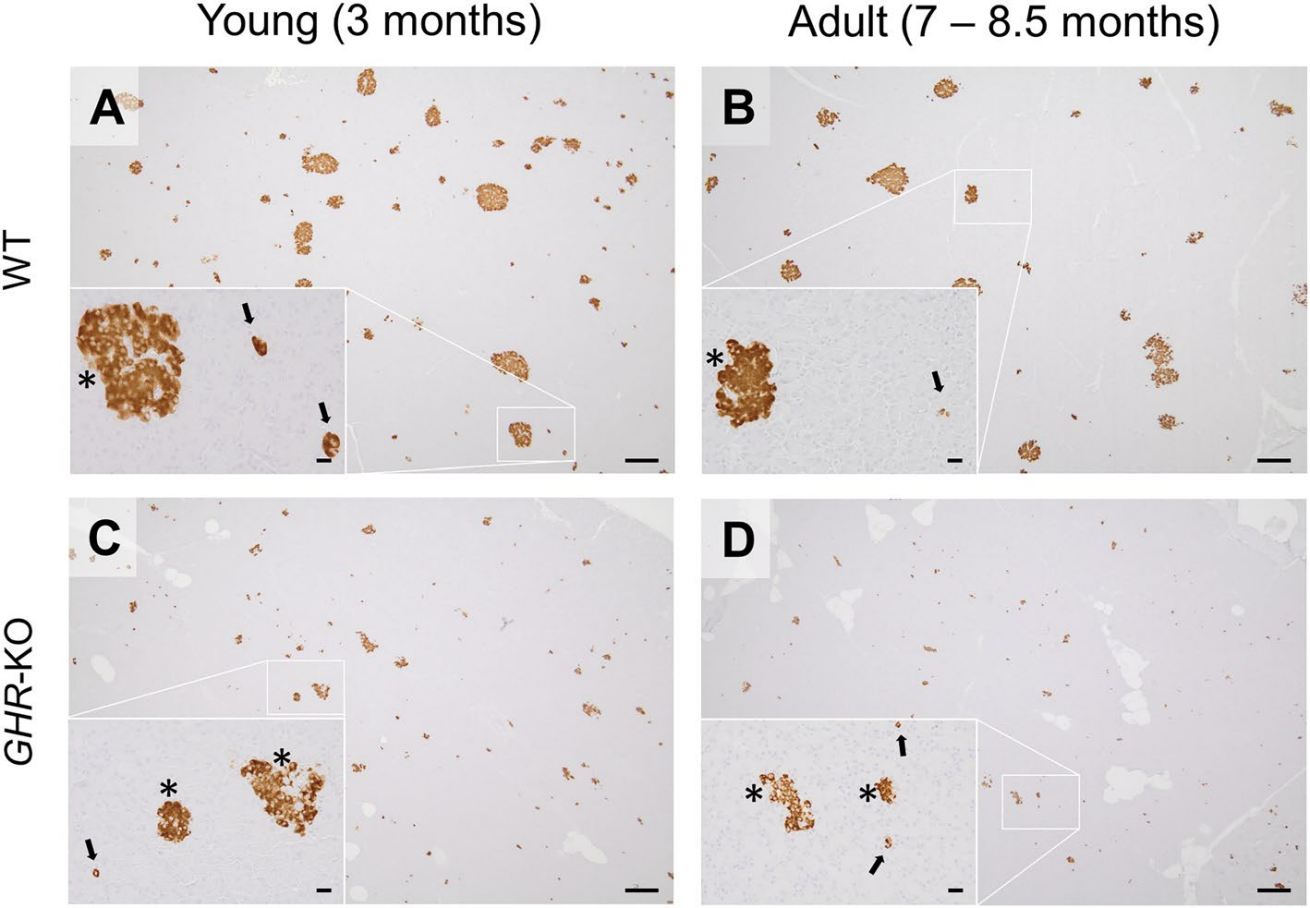
Immunohistochemical detection of insulin-positive cells in porcine pancreas. * indicate β-cells within clustered islet formation; arrows indicate isolated β-cells. Bars = 200 µm and 20 µm in insets

According to the reduction in body weight, the total volumes of the pancreata (V_(Pan)_) were also significantly reduced (Fig. 2B; p < 0.0001 for the effect of group) by 69% at young (24.9 ± 2.2 cm^3^ in *GHR*-KO vs. 80.4 ± 5.0 cm^3^ in WT pigs) and 58% at adult age (67.6 ± 7.3 cm^3^ in *GHR*-KO vs. 159.3 ± 11.3 cm^3^ in WT pigs). Nevertheless, pancreatic volume of *GHR*-KO pigs was proportionally reduced when corrected for body weight (Fig. 2C). The volume density of β-cells in the pancreas (V_V(β-cell/Pan)_) was significantly reduced in *GHR*-KO pigs in comparison to WT controls (Fig. 2D; Fig. 3; p = 0.0018 for the effect of group) by ~ 42% at young and ~ 39% at adult age. The total β-cell volume (V_(β-cell, Pan)_) was also significantly lower in *GHR*-KO than WT pigs (Fig. 2E; p < 0.0001 for the effect of group) and showed an increase with age regardless of the genotype (p = 0.001 for the effect of age). When corrected for body weight, the total β-cell volume (V_((β-cell, Pan)/BW)_; syn. β-cell mass), was reduced in *GHR*-KO pigs (Table S1; p < 0.0001 for the effect of group). While it decreased with age in WT pigs, values were not affected by age in *GHR*-KO pigs (Table S1; p = 0.0058 for the interaction group*age).

### *GHR*-KO pigs display an increased proportion of isolated β-cells in the pancreas, especially in adulthood

The volume density of isolated β-cells in the pancreas (V_V(isoβ-cell/Pan)_) was generally higher in young than in adult animals (Fig. 2F; Fig. 3; Table S1; p = 0.016 for the effect of age). Regardless of age, *GHR*-KO pigs displayed an increase in volume density of isolated β-cells in the pancreas (V_V(isoβ-cell/Pan)_) in comparison to WT controls (p = 0.0009 for the effect of group). The increased proportion of isolated β-cells in the pancreas of *GHR*-KO pigs appeared most prominent in adulthood (+ 97% in comparison with WT controls), and less pronounced at young age (+ 28% in comparison with WT controls).The total volume of isolated β-cells in the pancreas (V_(isoβ-cell, Pan)_) was generally reduced in *GHR-*KO pigs (Fig. 2G; p = 0.0126 for the effect of group). This was less pronounced at adult age when *GHR*-KO pigs` values almost narrowed the ones observed in WT controls (304.1 ± 48.6 mm^3^ vs. 335.6 ± 18.6 mm^3^). In contrast, the total volume of isolated β-cells in the pancreas of young *GHR*-KO pigs was reduced by more than 60% compared to young WT controls (115.9 ± 13.0 mm^3^ vs. 297.8 ± 21.2 mm^3^). At young age, the relative total volume of isolated β-cells in the pancreas (V_((isoβ-cell, Pan)/BW)_) was undistinguishable between *GHR*-KO and WT pigs, when corrected for body weight (6.5 ± 1.0 mm^3^/kg vs. 6.7 ± 0.9 mm^3^/kg). A decrease in the total volume of isolated β-cells in relation to body weight was observed in adult WT pigs (Table S1; p = 0.0208 for the interaction group*age).

### Despite reduced pancreatic insulin secretion, *GHR*-KO pigs show undisturbed glucose tolerance

**Fig. 4:**
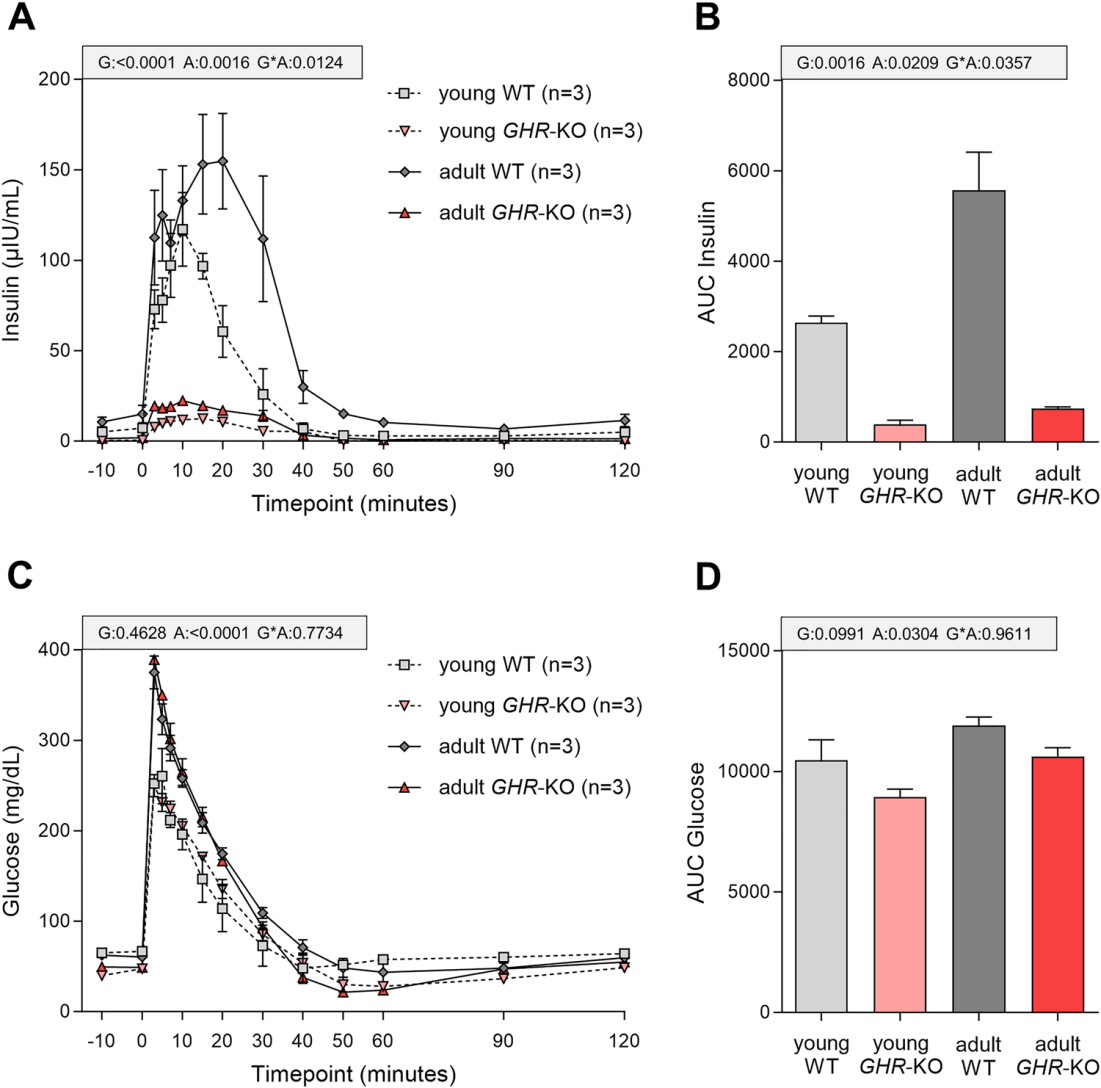
Intravenous glucose tolerance tests in *GHR*-KO and wild-type (WT) pigs aged 3 months (young) and 8.5 months (adult). *GHR*-KO pigs showed a significantly reduced insulin secretion in response to glucose administration at time point 0 (**A**–**B**). Glucose tolerance was not different between *GHR*-KO pigs and WT controls of the respective age (**C**–**D**). Data are mean ± SEM. Abbreviations: G = group, A = age, G*A = interaction group*age

To assess the consequences of a reduced β-cell volume and volume proportion of β-cells in the pancreas of *GHR*-KO pigs on metabolism in vivo, we evaluated the insulin secretion capacity and glucose tolerance by intravenous glucose tolerance tests. Compared with WT pigs, *GHR*-KO pigs showed a markedly decreased secretion of insulin in response to glucose bolus injection (Fig. 4A; p < 0.0001 for the effect of group). In both groups, the insulin secretion increased with age (p = 0.0016 for the effect of age), but this effect was less pronounced in *GHR*-KO than in WT pigs, which displayed a higher peak and prolonged insulin secretion in adulthood (p = 0.0124 for the interaction group*age). Corresponding group- and age-related differences were observed for AUC insulin (Fig. 4B). Despite reduced insulin secretion in *GHR*-KO pigs, no difference in glucose clearance was observed (Fig. 4C; p = 0.4628 for the effect of group). In both groups, higher maximum glucose levels (Fig. 4C; p < 0.0001 for the effect of age) and AUC glucose values (Fig. 4D; p = 0.0304 for the effect of age) were observed in adult animals, indicating an overall decrease in glucose tolerance with age regardless of genotype.

## Discussion

Our study highlights the advantages of genetically tailored large animal models in the field of metabolic research [30] and the *GHR*-KO pig as a model for GH insensitivity in particular. We were able to show that GH insensitivity impedes β-cell maturation and function and that the *GHR*-KO pig resembles the phenotype of human Laron syndrome (LS) patients who display increased insulin sensitivity despite obesity.

Already in 1980, it was observed that GH is able to mediate insulin secretion in vivo after injection into rats as well as in cell culture experiments on isolated islets of Langerhans [2]. A proliferative effect of GH was shown by Nielsen et al*.* [31], culturing isolated rat β-cells in the presence or absence of human recombinant GH. The implementation of transgenic mouse models further clarified the dependence of normal β-cell development and function on GH signaling. An increase in relative pancreas weight was observed in 15-week-old giant bovine GH overexpressing mice [32]. Furthermore, the expression of human GH under the mouse insulin promoter led to an increased β-cell proliferation in transgenic mice [33]. In contrast, *Ghr*-KO mice displayed a diminished pancreatic islet size [3] and islet cell mass in relation to body weight [11]. This resulted from decreased islet cell proliferation in the absence of functional GH stimulation [3]. Notably, islet-specific expression of an insulin-like growth factor 1 transgene restored islet cell mass through cell hypertrophy in global *Ghr*-KO mice, improving the insulin secretion capacity and thereby glucose tolerance [11].

Performing the first quantitative histomorphological investigation of the pancreas in a large animal model for GH insensitivity, our findings support the strong dependence on functional GH signaling for β-cell proliferation. GH insensitivity in *GHR*-KO pigs was associated with a reduction of the total β-cell mass (V_(β-cell, Pan)_), which appeared in proportion to the reduction in body weight. Due to disrupted GH signaling, *GHR*-KO pigs displayed a reduced volume density of β-cells in the pancreas (V_V(β-cell/Pan)_). Isolated β-cells were defined as single insulin-positive cells and small clusters of insulin-positive cells not belonging to established islets and are also referred to as extra-insular β-cells in the literature [34, 35]. Experiments with purified isolated rat β-cells observed a decreased insulin secretion capacity in comparison with intact islets of Langerhans [36]. In transgenic porcine models for diabetes research, the abundance of isolated β-cells was interpreted as a marker for pancreatic islet neogenesis [21] from progenitor cells [37], recapitulating embryonic development [38]. An increased abundance of non-islet β-cells is commonly observed in neonatal rats, while they are supposed to mature into fully developed islets of Langerhans during pancreas aging [39]. Therefore, an increased occurrence of isolated β-cells may be interpreted as an indicator for an immature stage. This is supported by their increased abundance in young WT pigs and decrease with age, while their increased abundance and proportion in the pancreas remained elevated in adult *GHR*-KO pigs in the absence of GH signaling.

While not directly assessed, a reduction in β-cell mass can be expected for LS patients as a study comparing 27 patients with 35 healthy relatives observed significantly reduced fasting insulin levels and HOMA-β scores [10]. Nevertheless, LS patients were able to maintain glucose tolerance even despite a decreased secretion capacity for insulin [10]. Liu et al. [3] investigated the β-cell mass of 10- to 11-month-old *Ghr*-KO mice and found a ~ 80% reduction of absolute β-cell mass and a 50% reduction when corrected for body weight in comparison to controls. Together with the observations of a reduced insulin secretion capacity and failure to clear the administered amount of glucose during intraperitoneal glucose tolerance tests [11], this indicates that rodent β-cells are even more dependent on GH action than those of pigs and humans. While *Ghr*-KO mice can be considered glucose intolerant [3, 11], they nevertheless showed fasting hypoglycemia, suggesting insulin hypersensitivity [3] as in human LS patients [10].

Increased insulin sensitivity in the absence of functional GH signaling is commonly attributed to the lack of direct insulin-antagonizing effects of GH, as well as metabolic alterations resulting from GH insensitivity. Excess GH decreases insulin receptor (IR) expression, while increased IR levels and improved intracellular signaling are observed in GH insensitivity [5, 40]. In *Ghr*-KO mice, Chhabra et al*.* [40] demonstrated that the absence of GH receptor-mediated signal transducer and activator of transcription 5 (STAT5) activation improves insulin sensitivity and also reduces glucose output from the liver. GH is a potent mediator of lipolysis, stimulating the release of NEFAs, especially in the fasting state [41]. Consequently, states of GH insensitivity are characterized by increased accumulation of adipose tissue but decreased NEFA levels, which improves hepatic insulin sensitivity [42].

Pigs do not only resemble humans in regard to their size and body weight, but also share major similarities in pancreatic islet architecture, vascularization, β-cell mass and transcriptome, as well as insulin structure, making the pig a favorable animal model to study alterations in pancreatic islet function and pathophysiology [30, 43]. Another important similarity between pig and human pancreata can be found in pancreatic development, with particular emphasis on endocrine differentiation, where pigs have been shown to resemble humans more closely than murine models [44]. In line with this, *GHR*-KO pigs displayed decreased basal insulin levels and insulin secretion in response to glucose bolus infusion, while glucose tolerance remained preserved. Regardless of genotype, we observed an increase in fasting insulin, insulin secretion and glucose rise in ivGTTs with age. This is consistent with the results in healthy human subjects, in whom insulin sensitivity decreased physiologically with age [45, 46] while insulin secretion increased in a compensatory manner [47].

In conclusion, our results demonstrate that the *GHR*-KO pig is a suitable large animal model for GH insensitivity, showing a reduction in total β-cell mass and β-cell volume density in the pancreas. The *GHR*-KO pig particularly resembles the phenotype of Ecuadorian LS patients, where a preserved glucose tolerance despite decreased insulin secretion capacity results from an increased insulin sensitivity despite obesity.

## Supplementary Information

Below is the link to the electronic supplementary material.

Supplementary file1 (PDF 158 KB)—Supplementary Table 1 Growth parameters and quantitative stereological data of *GHR*-KO and WT control pigs at young and adult age

## Data Availability

Data availability upon request
