# Supplementary material for: Decreased β-cell volume and insulin secretion but preserved glucose tolerance in a growth hormone insensitive pig model
Source: Pituitary. 2024 Jul 3;27(5):567–76. doi: 10.1007/s11102-024-01424-w (PMC11513746; doi:10.1007/s11102-024-01424-w)
Supplement: Supplementary file 1 — Supplementary file1 (PDF 158 KB)—Supplementary Table 1 Growth parameters and quantitative stereological data of GHR-KO and WT control pigs at young and adult age [file 11102_2024_1424_MOESM1_ESM.pdf]

Parameter	young WT	young *GHR*-KO	adult WT	adult *GHR*-KO	Group	Sex	Age	Group*Sex	Group*Age
	Mean ± SEM				p-value				
Body weight (BW) (kg)	45.6 ± 3.2	18.2 ± 0.9	126.2 ± 4.9	48.7 ± 3.4	<0.0001	0.4975	<0.0001	0.4876	<0.0001
Pancreas weight (Pa) (g)	86.0 ± 5.3	26.6 ± 2.3	170.4 ± 12.1	72.3 ± 7.8	<0.0001	0.6453	<0.0001	0.9264	0.0879
Rel. Pa weight (% of BW)	0.191 ± 0.012	0.148 ± 0.015	0.134 ± 0.006	0.150 ± 0.009	0.222	0.1754	0.0997	0.2745	0.0086
$V_{(Pan)}$ [cm^3]	80.4 ± 5.0	24.9 ± 2.2	159.3 ± 11.3	67.6 ± 7.3	<0.0001	0.6453	<0.0001	0.9264	0.0879
$V_{(Pan/BW)}$ [cm^3/kg]	1.78 ± 0.11	1.38 ± 0.14	1.26 ± 0.06	1.40 ± 0.09	0.222	0.1754	0.0997	0.2745	0.0086
$Vv_{(\beta\text{-cell/Pan})}$ [%]	2.08 ± 0.30	1.21 ± 0.19	1.83 ± 0.23	1.12 ± 0.12	0.0018	0.8646	0.5685	0.3842	0.4682
$V_{(\beta\text{-cell, Pan})}$ [mm^3]	1667.0 ± 254.7	288.3 ± 27.1	2789.0 ± 191.1	741.4 ± 65.0	<0.0001	0.7173	0.001	0.1931	0.2702
$V_{((\beta\text{-cell, Pan})/BW)}$ [mm^3/kg]	35.9 ± 3.6	16.1 ± 1.8	22.4 ± 2.0	16.1 ± 2.2	<0.0001	0.4534	0.0393	0.1452	0.0058
$Vv_{(iso\beta\text{-cell/Pan})}$ [%]	0.376 ± 0.032	0.482 ± 0.064	0.216 ± 0.017	0.426 ± 0.035	0.0009	0.5708	0.016	0.844	0.2311
$V_{(iso\beta\text{-cell, Pan})}$ [mm^3]	297.8 ± 21.2	115.9 ± 13.0	335.6 ± 18.6	304.1 ± 48.6	0.0126	0.5084	0.0107	0.6635	0.0293
$V_{((iso\beta\text{-cell, Pan})/BW)}$ [mm^3/kg]	6.7 ± 0.9	6.5 ± 1.0	2.7 ± 0.1	6.2 ± 0.7	0.032	0.9009	0.0153	0.6078	0.0208
